# Menisoxoisoaporphine A, a novel oxoisoaporphine alkaloid from Menispermi Rhizoma, inhibits inflammation by targeting PDE4B

**DOI:** 10.3389/fphar.2024.1505116

**Published:** 2024-12-03

**Authors:** Xin Qiao, Xiaojuan Cao, Shuang Xu, Cunlin Wang, Rui Guo, Xiaojuan Yao, Qiong Zhang

**Affiliations:** ^1^ School of Pharmacy, Shanxi Medical University, Taiyuan, China; ^2^ Medicinal Basic Research Innovation Center of Chronic Kidney Disease, Ministry of Education, Shanxi Medical University, Taiyuan, China; ^3^ Shanxi Provincial Key Laboratory of Drug Synthesis and Novel Pharmaceutical Preparation Technology, Shanxi Medical University, Taiyuan, China; ^4^ Academy of Medical Sciences, Shanxi Medical University, Taiyuan, China

**Keywords:** menisoxoisoaporphine A, inflammation, PDE4B, cAMP-PKA, NF-κB

## Abstract

**Background:**

Dysregulated and excessive inflammatory reactions can lead to tissue damage, which is the underlying cause of most human diseases. Menisoxoisoaporphine A (MA), a novel oxoisoaporphine alkaloid, was obtained from Menispermi Rhizoma, a traditional Chinese medicinal herb used in the treatment of inflammatory conditions in clinical practice. This suggests that MA has very promising potential for the development of anti-inflammatory therapeutics. Hence, this study aims to investigate the anti-inflammatory effects and underlying mechanisms of MA.

**Method:**

The anti-inflammatory effects of MA were evaluated in lipopolysaccharide (LPS)-induced mouse macrophage RAW264.7 cells. Its underlying mechanisms were explored through RNA sequencing and Western blotting. The binding modes and interactions sites between MA and phosphodiesterase 4B (PDE4B) were predicted using molecular docking and validated by molecular dynamics simulation.

**Results:**

MA treatment significantly reduced LPS-induced morphological changes, inflammatory cytokine relesae, and proinflammatory genes expression in RAW264.7 cells compared to the LPS-induced controls. Transcriptome sequencing analysis suggested that PDE4B might be a key target for MA to exert its therapeutic effect. Mechanismly, MA directly acted on Tyr405 site of PDE4B, thus leading to a sustained elevation of the cyclic adenosine monophosphate (cAMP) levels, which subsequently inactivated NF-κB signaling pathway by phosphorylating protein kinase A (PKA). MA inhibited the NF-κB-mediated inflammatory response depending on PDE4B.

**Conclusion:**

MA, a natural and novel compound, exerted anti-inflammatory effects in LPS-induced RAW264.7 macrophage cells. It demonstrated a strong binding ability to the Tyr405 sites of PDE4B, thereby inhibiting NF-κB signaling pathway by regulating the cAMP-PKA axis. Elucidating the interaction between MA and PDE4B holds significant potential for the advancement of innovative therapeutic strategies aimed at inflammatory diseases. By strategically modulating this interaction, it may be feasible to achieve more precise regulation of inflammatory responses, thereby offering promising therapeutic benefits for conditions such as rheumatoid arthritis, asthma, and inflammatory bowel disease.

## 1 Introduction

Inflammation is an inherent physiological reaction of the body to particular stimuli, serving as a defensive mechanism (Zarrin et al., 2021). An excessive inflammatory response is a hallmark of many acute or chronic diseases, such as in-flammatory bowel disease, rheumatoid arthritis, atherosclerosis, and cancer (Herrero-Cervera et al., 2022). In recent years, inflammatory diseases have become an intractable public health problem worldwide due to their increasing global incidence and prevalence (Ng et al., 2017). The pathogenesis of inflammatory diseases is complex, involving multiple signaling mechanisms in inflammatory responses, among which the NF-κB pathway is one of the best-characterized signaling pathways (Yu et al., 2020; Zhu et al., 2018). Currently, standard clinical treatments for inflammation-related diseases comprise corticosteroids, selective cyclooxygenase-2 inhibitors, and non-steroidal anti-inflammatory drugs. However, prolonged administration is linked to significant adverse reactions (Han et al., 2023). Thus, new anti-inflammatory agents are desperately needed.

Menispermi Rhizoma (MR), known as Beidougen in Chinese, is the dried rhizome of *Menispermum dauricum* DC. (Chinese Pharmacopoeia Commission, 2020). It has been widely used in traditional Chinese medicine (TCM) for the treatment of sore throat and rheumatic arthralgia (Anon, 1977). The chemical constituents of MR mainly include alkaloids, phenolic acids, quinones, and polysaccharide, with alkaloids serving as the predominant pharmacologically active compounds (Zhai et al., 2023). Capsulae Menispermi and BeiDouGenPian, mainly composed of total alkaloids of MR, have been used clinically in China for the treatment of inflammatory diseases including pharyngitis, bronchitis, and tonsillitis, thus suggesting the alkaloids of MR with very promising potential for the development of anti-inflammatory therapeutics (Wu and Jin, 2007; Huang et al., 2007; Sun and Wang, 2009). In recent years, investigations into the anti-inflammatory activity of MR have primarily centered on the total alkaloids of MR and some of its major components, especially bisbenzylisoquinoline and morphine alkaloids, with less attention given to new compounds (Yao et al., 2023). Currently, the limited variety of alkaloids studied in anti-inflammatory research cannot fully explain the significant pharmacological basis for the anti-inflammatory activity of MR. Menisoxoisoaporphine A (MA, 6-(isopentylamino)-4,5,9-trimethoxy-*7H*-dibenzo [*de*,*h*]quinolin-7-one) is a new oxoisoaporphine alkaloid extracted from MR by our group, demonstrating significant anti-inflammatory potential (Guo et al., 2024). Our study represents the first investigation into the anti-inflammatory effects and underlying mechanisms of MA, which are crucial for the development of pharmacological agents targeting PDE4B signaling pathways.

Phosphodiesterases 4 (PDE4), which are encoded by four genes (*Pde4a*, *Pde4b*, *Pde4c*, and *Pde4d*), comprise a superfamily of enzymes responsible for the hydrolysis of the second messengers cyclic adenosine monophosphate (cAMP) and cyclic guanosine monophosphate (cGMP). These molecules are integral to the regulation of inflammatory processes (He et al., 2020a; Wakabayashi et al., 2022). PDE4B is a well-validated drug target for regulating the inflammatory response; it is the dominant PDE4 in macrophages, and mice deficient in PDE4B have a diminished ability to respond to inflammatory stimuli (Wade et al., 2019; Li et al., 2019). PDE4B inhibitors have been documented to exhibit therapeutic efficacy in the treatment of allergic disorders (Jin et al., 2010). PDE4B is also significantly involved in the regulation of the inflammatory response in microglia (Pearse and Hughes, 2016). Accumulating evidence has demonstrated that elevation of intracellular cAMP caused by PDE4 inhibitors leads to inhibition of the production of pro-inflammatory cytokines such as IL-6 and TNF-α (Hedde et al., 2017). Protein kinase A (PKA) serves as a significant mediator of cyclic adenosine monophosphate (cAMP) signaling. Upon binding of cAMP to the regulatory subunit of PKA, the enzyme is activated and undergoes phosphorylation, thereby inhibiting the activation of downstream signaling pathways, such as the NF-κB signaling pathway (Zhu et al., 2021).

In this work, we aimed to identify candidate molecules crucial for the protective effects of MA on lipopolysaccharide (LPS)-induced inflammation in RAW264.7 cells through RNA sequencing (RNA-seq) analysis. Among numerous regulated proteins, PDE4B has been previously identified as closely linked to inflammation and may serve as a primary target through which MA exerts its anti-inflammatory effects. We found that MA plays a role by regulating the PDE4B-cAMP-PKA-NF-κB signaling pathway. MA directly acts on the Tyr405 site of PDE4B, and it inhibits the NF-κB-mediated inflammatory response, depending on PDE4B. To sum up, these findings not only imply that MA possesses significant anti-inflammatory activity but also suggest that MA is a novel PDE4B inhibitor for the first time.

## 2 Materials and methods

### 2.1 Reagents

Indomethacin (IM, purity ≥98%) was purchased from Aladdin (Shanghai, China), and lipopolysaccharide (LPS) was obtained from Biosharp (Guangzhou, China). Secondary antibodies, including HRP-conjugated goat anti-rabbit antibodies (BS13278) and HRP-conjugated goat anti-mouse antibodies (BS12478), were sourced from Bioworld (Nanjing, China). PDE4B-IN-3 (IN-3, purity ≥98%) was purchased from MedChemExpress (Shanghai, China).

### 2.2 Isolation of MA

**FIGURE 1:**
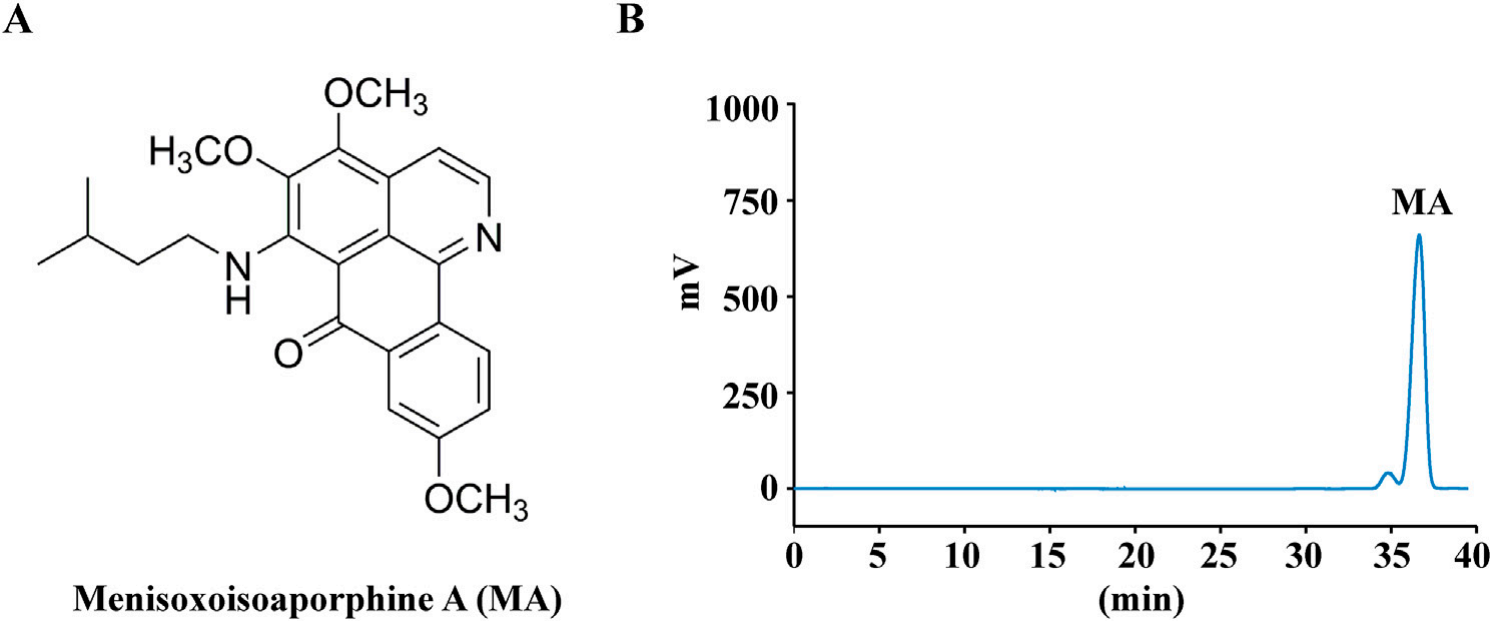
Chemical structures and HPLC analysis of Menisoxoisoaporphine A (MA). **(A)** The structures of the determined MA. **(B)** HPLC chromatogram of MA recorded at 260 nm.

Menisoxoisoaporphine A (MA, purity ≥98%) was extracted and isolated by our research group (Guo et al., 2024). Briefly, milled rhizomes of *Menispermum dauricum* DC. (100 kg) were extracted with 95% ethanol (50 L × 3, 4 h each) to obtain the crude extract (30 kg). The alcohol extract (30 kg) was dissolved in acid, precipitated with alkali, and subsequently extracted with dichloromethane to yield a total alkaloid extract (2.4 kg). The total alkaloids were isolated systematically and purified using silica gel column chromatography as well as preparative and semi-preparative high-performance liquid chromatography. MA (25 mg, t_R_ 36.6 min) was purified by preparative HPLC with the mobile phase 65% MeOH/H_2_O (*v*/*v*). The structures of MA was shown in Figure 1A. Figure 1B displays the HPLC chromatogram of MA detected at 260 nm.

### 2.3 Cell culture

Mouse peritoneal macrophage cells RAW264.7, sourced from the Cell Bank of the Chinese Academy of Sciences in Shanghai, were maintained in Roswell Park Memorial Institute 1640 medium (RPMI1640, KeyGen, Nanjing, China), supplemented with 10% (*v*/*v*) fetal bovine serum (FBS, Procell, China), penicillin (100 U·mL^-1^), and streptomycin (100 μg·mL^-1^) in a humidified 5% CO_2_ incubator at 37°C.

### 2.4 Cell viability

Cell viability were determined by tetrazolium compound 3-[4,5-dimethylthiazol-2-yl]-2,5-diphenyltetrazolium bromide (MTT) assay. Briefly, 6 × 10^3^ cells were seeded in a 96-well plate. After 12 h, the cells were exposed to LPS at a concentration of 1 μg·mL^-1^, along with varying concentrations of MA, for a duration of 24 h. Then, the cells were incubated with MTT reagent for another 4 h. After that, the media with the reagent was carefully discarded, and 100 μL of dimethyl sulfoxide (DMSO, Solarbio, Beijing, China) was added to each well in order to dissolve the formazan crystals. The absorbance of each well was quantified using a microplate reader (SpectraMax 190, Molecular Devices) set to a wavelength of 570 nm. Cell viability was calculated as follows: Cell viability (%) = (OD_treatment group_ - OD_blank group_/OD_control_ - OD_blank group_) × 100%. Experiments were performed at least for 3 times using 6 replicates for each sample.

### 2.5 Cell morphology

Briefly, cells were seeded into 6-well plates at a density of 6 × 10^3^ per well, with a final volume of 2 mL. After the cells became adherent, the original medium was removed, and cells were stimulated with 1 μg·mL^-1^ LPS in the presence of MA (3, 6, 12 μM) or IM (10 μM) for 24 h. Cell morphology was examined utilizing an inverted microscope (DMi8, Leica, Germany).

### 2.6 Measurement of IL-6, TNF-α and NO

The concentrations of IL-6 and TNF-α in cell culture supernatants were quantified through the utilization of Enzyme-linked immunosorbent assay (ELISA) kits, following the guidelines provided by the manufacturers (MultiSciences Biotech, Hangzhou, China). Nitric oxide (NO) production was quantified utilizing the Total Nitric Oxide Assay Kit (Beyotime, Shanghai, China) in accordance with the manufacturer’s instructions.

### 2.7 Quantitative real-time polymerase chain reaction (RT-PCR)

Total RNA extraction was conducted using TRIzol reagent (Vazyme, Nanjing, China), followed by cDNA synthesis using the HiScript II reverse transcriptase kit (Vazyme, Nanjing, China). Quantitative PCR was performed using ChamQ SYBR qPCR Master Mix (Vazyme, Nanjing, China) in accordance with the manufacturer’s guidelines on a real-time-PCR system (QuantStudio 3, Life Tech, United States). The mRNA levels of the target genes were normalized to β-actin and quantified using the 2^−ΔΔCt^ method. Primer sequences were shown in Table 1.

**TABLE 1:** Primer sequences used in this study.

Species	Gene	Sequences (5′-3′)
Mouse	*β-actin*	Forward: GGC​TGT​ATT​CCC​CTC​CAT​CG
		Reverse: CCA​GTT​GTA​ACA​ATG​CCA​TGT
Mouse	*Il-6*	Forward: TAC​CAC​TTC​ACA​AGT​CGG​AGG​C
		Reverse: CTG​CAA​GTG​CAT​CAT​CGT​GTT​C
Mouse	*Nos2*	Forward: CCTGGTZCGGGCATTGCT
		Reverse: CGGCACCCAAACACCAA
Mouse	*Tnf-α*	Forward: GCC​AAC​GGC​ATG​GAT​CTC​AA
		Reverse: TCT​TGA​CGG​CAG​AGA​GGA​GG
Mouse	*Pde4b*	Forward: GAC​TAC​ATT​GTC​CAT​CCA​CTG​TG
		Reverse: CTT​GAG​CAT​CCG​GTT​GAA​CCA
Mouse	*Igf2r*	Forward: GGG​AAG​CTG​TTG​ACT​CCA​AAA
		Reverse: GCA​GCC​CAT​AGT​GGT​GTT​GAA
Mouse	*Slc6a12*	Forward: GGT​CCC​TGA​GGA​AGG​AGA​GAT
		Reverse: GGG​GAT​GAA​GAA​AGC​TCC​ACC
Mouse	*Itgb7*	Forward: ACC​TGA​GCT​ACT​CAA​TGA​AGG​A
		Reverse: CAC​CGT​TTT​GTC​CAC​GAA​GG
Mouse	*Tlr9*	Forward: ATG​GTT​CTC​CGT​CGA​AGG​ACT
		Reverse: GAG​GCT​TCA​GCT​CAC​AGG​G
Mouse	*Nox1*	Forward: GGT​TGG​GGC​TGA​ACA​TTT​TTC
		Reverse: TCG​ACA​CAC​AGG​AAT​CAG​GAT
Mouse	*Mmp12*	Forward: CTG​CTC​CCA​TGA​ATG​ACA​GTG
		Reverse: AGT​TGC​TTC​TAG​CCC​AAG​AAC
Mouse	*Abca1*	Forward: AAA​ACC​GCA​GAC​ATC​CTT​CAG
		Reverse: CAT​ACC​GAA​ACT​CGT​TCA​CCC
Mouse	*Cd80*	Forward: ACC​CCC​AAC​ATA​ACT​GAG​TCT
		Reverse: TTC​CAA​CCA​AGA​GAA​GCG​AGG
Mouse	*Il-11*	Forward: TGT​TCT​CCT​AAC​CCG​ATC​CCT
		Reverse: CAG​GAA​GCT​GCA​AAG​ATC​CCA
Mouse	*Tnfrsf9*	Forward: CGT​GCA​GAA​CTC​CTG​TGA​TAA​C
		Reverse: GTC​CAC​CTA​TGC​TGG​AGA​AGG

### 2.8 Transcriptome analysis

Transcriptome analysis of RAW264.7 cells was performed using the RNA-sequencing method (RNA-seq) by BGI Technology Services Co., Ltd. (Shenzhen, China). In brief, total RNA was extracted utilizing Trizol (Invitrogen, Carlsbad, CA, United States). The concentration of total RNA was determined using the Equalbit RNA BR Assay Kit (Invitrogen, MA, United States). The qualified RNA samples were used for single-strand circle DNA (ssCir DNA) library construction. The sequencing substrate DNA nanoballs (DNBs) were generated from ssCir DNA by rolling circle amplification. Then, the DNBs were then incorporated into the structured nanoarray through the utilization of high-density DNA nanochip technology. Finally, library sequencing was performed using a combinatorial probe-anchor synthesis (cPAS)-based sequencer. To acquire clean data, the raw data underwent filtration utilizing using the SOAPnuke Toolkit software (v1.5.6). Then, all the clean data were analyzed on the Dr. Tom network platform (https://biosys.bgi.com/) provided by BGI. Clean data were mapped to the reference genome via HISAT2 (v2.1.0) and aligned to the reference genome set using Bowtie2 (v2.2.5). RSEM (v1.3.1) was used to calculate the expression of genes and transcripts. Fragments per kilobase of transcript per million fragments mapped (FPKM) was utilized as a metric for gene expression levels. DEseq2 (v1.4.5) was employed to detect differentially expressed genes (DEGs) with a Q-value ≤0.05. Volcano plots were acquired based on DEGs with |log2-fold change (FC)| ≥ 1.5. Venn diagrams and hierarchical clustering were generated using the DEGs between control-model and model-MA to obtain common DEGs. Three biological replicates were prepared in each group.

### 2.9 Western blot analysis

Cells were lysed with ice-cold radio immunoprecipitation assay (RIPA) buffer (Beyotime, Hangzhou, China) containing protease inhibitor (Beyotime, Hangzhou, China) and phosphatase inhibitor (Beyotime, Hangzhou, China). Total protein concentration was determined with a bicinchoninic acid (BCA) protein assay kit (Beyotime, Hangzhou, China). All proteins were resolved in 10% SDS-polyacrylamide gel electrophoresis (SDS-PAGE) gels and transferred to polyvinylidene fluoride (PVDF) membranes (Millipore, United States). The membrane was blocked for 2 h at room temperature using a 5% skim milk solution prior to incubation with the primary antibody at 4°C overnight. Then secondary antibodies were incubated for 2 h at room temperature. Immunoreactive bands were visualized by using chemiluminescence (Elabscience, Wuhan, China), followed by quantitative analysis using ImageJ software (NIH, United States). Primary antibodies were shown in Supplementary Table S1.

### 2.10 Cell transfection

To silence the expression of PDE4B, a *Pde4b* small interfering RNA (siRNA) and a control siRNA were chemically synthesized by GenePharma Co., Ltd. (Shanghai, China) and transfected into RAW264.7 cells using Lipofectamine 2000 Transfection Reagent (Invitrogen, CA, United States) according to the manufacturer’s instructions. The siRNA sequences were shown in Table 2. For PDE4B overexpression, the pIRES2-EGFP-PDE4B plasmid and empty pIRES2-EGFP plasmid were constructed by Public Protein/Plasmid Library (PPL, China).

**TABLE 2:** siRNA sequences used in the study.

Gene	Directions	Sequence (5′-3′)
siRNA Control	sense	UUC​UCC​GAA​CGU​GUC​ACG​UTT
	antisense	ACG​UGA​CAC​GUU​CGG​AGA​ATT
siRNA *Pde4b* #1	sense	CCA​GGA​AAC​AGA​CCU​ACA​UTT
	antisense	AUG​UAG​GUC​UGU​UUC​CUG​GTT
siRNA *Pde4b* #2	sense	GGC​UCA​UAC​AUG​CUU​UGA​UTT
	antisense	AUC​AAA​GCA​UGU​AUG​AGC​CTT
siRNA *Pde4b* #3	sense	GCG​ACA​UCU​UUC​AGA​AUC​UTT
	antisense	AGA​UUC​UGA​AAG​AUG​UCG​CTT
siRNA *Pde4b* #4	sense	GAC​GCU​UUG​UGU​GAU​UGA​UTT
	antisense	AUC​AAU​CAC​ACA​AAG​CGU​CTT

### 2.11 Molecular docking

The predicted structures of PDE4B were generated by AlphaFold. The protonation state of all the compounds was adjusted to pH 7.4, and their molecular structures were converted into three-dimensional representations using Open Babel (O'Boyle et al., 2011). AutoDock Tools (ADT3) were utilized for the preparation and parameterization of the receptor protein and ligands. The docking grid documents were created using AutoGrid of SiteMap, and the docking simulations were conducted using AutoDock Vina (1.2.0) (Eberhardt et al., 2021; Trott and Olson, 2010). The optimal pose was chosen for the analysis of interaction. Finally, the protein-ligand interaction diagram was produced using PyMOL.

### 2.12 Molecular dynamics simulations

The interaction between PDE4B and MA was studied by molecular dynamics (MD) simulation. The results of molecular docking was used as the initial wild-type conformation, and the tyrosine residue at position 405 (Tyr405) was replaced with alanine by SPDBV (Johansson et al., 2012) software to form an alanine mutant system. Gromacs 2023.1 (Mark et al., 2023) version was used for molecular dynamics simulation, and Amber ff14sb (He et al., 2020b) force field and GAFF (Wang et al., 2004) force field were used to parameterize proteins and small molecules, respectively. Using the TIP3P (Van Der Spoel and Van Maaren, 2006) water model, the two complex systems were explicitly solvated in a regular dodecahedron of appropriate periodic size. The steepest descent method was employed to optimize the energy of 0.1 ns in order to mitigate the presence of an unjustifiable energy barrier within the simulation. Na ions were added to both simulations to maintain the electric neutrality of the simulation system, and the energy of another 0.1 ns was minimized by conjugate gradient method. The system temperature was then raised from 0 K to 310 K for 1 ns in a constant temperature and volume (NVT) ensemble, controlled by a V-rescale thermostat. A 1 ns pre-balance was then performed in a constant temperature and constant pressure (NPT) system at 1 atm and 310 K, with a Berendsen pressure controller and a V-rescale thermostat controlling the temperature. Finally, the MD running time for each system was set to 100 ns. The entire simulation process was run in a NPT ensemble with a pressure of 1 atm and a temperature of 310 K, the step size was set to 2 fs, and the trajectory coordinates were recorded every 10 ps. In order to avoid the edge effect, the whole simulation process adopts periodic boundary conditions. After the simulation, VMD (Humphrey et al., 1996) software was used for visual analysis and trajectory animation, while the gmx_MMPBSA script (Valdés-Tresanco et al., 2021) was employed for combined free energy calculations.

### 2.13 Statistical analysis

All results were presented as the mean ± standard error of the mean (SEM) derived from a minimum of three distinct experiments. Statistical analyses were carried out utilizing the GraphPad software (v. 7.0.0, San Diego, CA, United States). The statistical methods of the Student’s t-test or one-way ANOVA were employed to compare groups within the study. Values of *p* < 0.05 indicated statistical significance.

## 3 Result

### 3.1 MA suppressed LPS-induced inflammation in RAW264.7 macrophages

**FIGURE 2:**
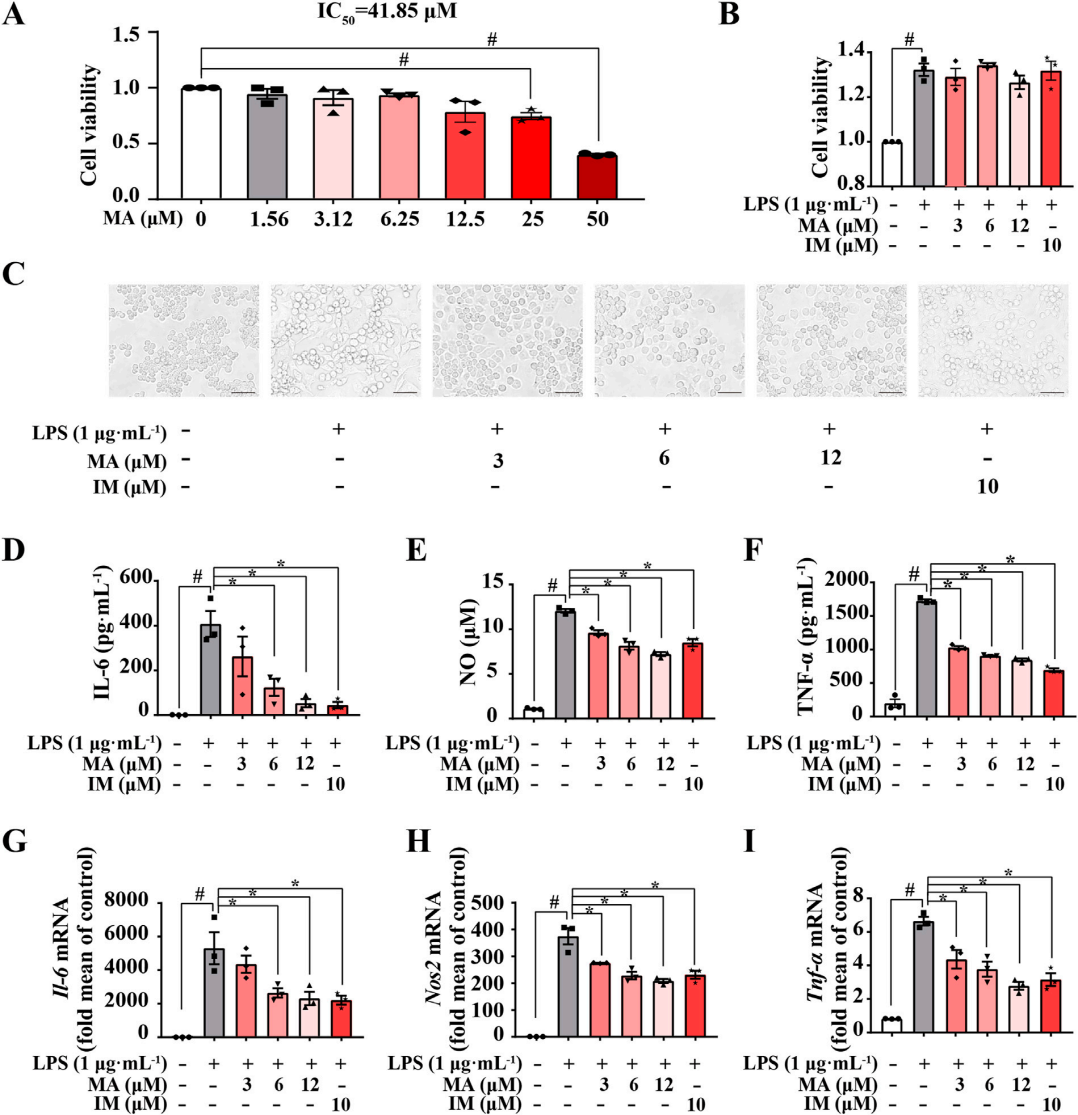
Menisoxoisoaporphine A (MA) suppressed LPS-induced inflammation in RAW264.7 macrophages. RAW264.7 cells were stimulated with 1 μg·mL^-1^ lipopolysaccharide (LPS), and treated with different concentrations of MA (0, 3, 6, 12 μM) or indomethacin (IM, 10 μM) concurrently. **(A)** Cell viability of MA was estimated on RAW264.7 cells by MTT assay. **(B)** Cell viability of the MA or IM was estimated on LPS-treated RAW264.7 cells by MTT assay. **(C)** Representative images of cell morphology after12 h treatment (Scale bars: 100 µm). **(D–F)** The expression of interleukin 6 (IL-6), nitric oxide (NO) and tumor necrosis factor-α (TNF-α) in the collected supernatant after 24 h treatment were determined by ELISA and a Nitric Oxide Kit. **(G–I)** The mRNA expressions level of *Il-6*, inducible nitric oxide synthase (*Nos2*) and *Tnf-α* in cells after 12 h treatment were detected by qRT-PCR. Each value was expressed as the means ± SEM (n = 3). ^#^ *p* < 0.05 vs*.* control group. *p < 0.05 vs*.* only LPS-treated group. IC_50_: half maximal inhibitory concentration.

To investigate whether MA can suppress LPS-induced inflammation in RAW264.7 macrophages, we preferentially tested the cytotoxicity of MA. The findings of the MTT assay indicated that MA exhibited no statistically significant cytotoxic effects on RAW264.7 cells at concentrations below 12.5 μM when compared to the control group (Figure 2A). Likewise, the selected concentrations of MA (3 μM, 6 μM, and 12 μM) were found to be non-cytotoxic for RAW264.7 macrophages challenged with LPS (Figure 2B). Indomethacin (IM; 10 μM) was employed as a positive control.

We then examined the *in-vitro* anti-inflammatory activities of MA in LPS-induced RAW264.7 macrophages. As shown in Figure 2C, RAW264.7 cells were small and round under normal conditions and became larger and extended pseudopods after LPS stimulation for 12 h. However, MA treatment markedly suppressed differentiation in RAW264.7 cells. We also found that pro-inflammatory macrophages upregulated the levels of inflammatory factors (IL-6, NO and TNF-α) after LPS stimulation, while MA effectively inhibited the release of these inflammatory factors (*p* < 0.05) (Figures 2D–F). To further assess the effects of MA on LPS-induced inflammatory response in RAW264.7, we examined mRNA expression for key inflammatory cytokine genes. As expected, MA significantly decreased the mRNA levels of *Il-6*, *Nos2*, and *Tnf-α* in a concentration-dependent manner (*p* < 0.05) (Figures 2G–I). These results indicated that MA could inhibit LPS-induced inflammation in RAW264.7 cells, suggesting its potential applications in the treatment of inflammatory diseases and the modulation of immune responses. Nonetheless, additional research is required to comprehensively elucidate the mechanisms underlying the action of MA.

### 3.2 RNA-seq analysis revealed the anti-inflammatory properties of MA were associated with PDE4B

**FIGURE 3:**
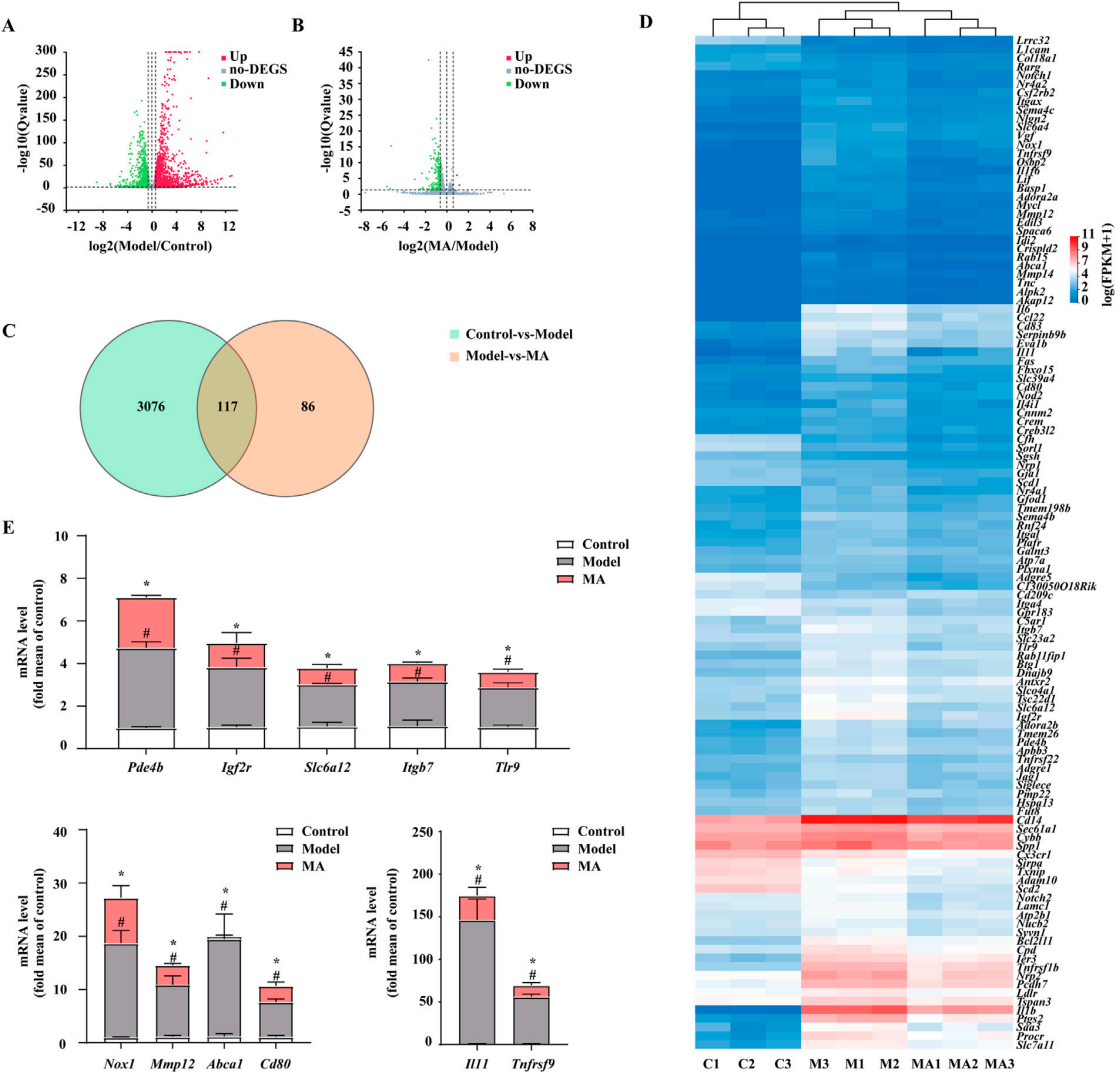
RNA-seq analysis revealed the anti-inflammatory properties of Menisoxoisoaporphine A (MA) were associated with PDE4B. RAW264.7 cells were stimulated with 1 μg·mL^-1^ lipopolysaccharide (LPS), and concurrently treated with MA (0, 12 μM) for 12 h. The cells were harvested for transcriptome profiling by RNA-seq. **(A)** Volcano plot showing DEGs between control vs*.* model groups. **(B)** Volcano plot showing DEGs between model vs*.* MA groups. **(C)** Venn diagram showing the co-differentially expressed genes (co-DEGs) between control vs*.* model and model vs*.* MA groups. **(D)** Heatmap showing the expression of 117 co-DEGs. **(E)** The mRNA relative expression of *Pde4b, Igf2r, Slc6a12, Itgb7, Tlr9, Nox1, Mmp12, Abca1, Cd80, Il11* and *Tnfrsf9* were validated by qRT-PCR in RAW264.7 cells. For the volcano plot, the abscissa represents the Log2 transformed fold-change, and the ordinate represents the -log10 transformed *p*-value. The green dots indicated the downregulated genes, the red dots indicated the upregulated genes and the grey dots indicated the non-DEGs. DEGs: differentially expressed genes (FPKM value, fold change ≥1.5 and *p* ≤ 0.05). Control (C1-C3): control normal group; Model (M1-M3): only LPS-treated group; MA (MA1-MA3): LPS and MA-treated group. Each value was expressed as the means ± SEM (n = 3). ^#^ *p* < 0.05: vs*.* control group. *p < 0.05: vs*.* only LPS-treated group.

To further probe the underlying inhibitory mechanisms against LPS, RNA-Seq was employed to evaluate transcriptome profiling in RAW264.7 cells. According to the established criteria of *q*-value ≤0.05 and log2-|fold change| ≥ 1.5, a total of 3,193 differentially expressed genes (DEGs) were detected in the comparison between the control and model groups. Among these DEGs, 1706 were found to be upregulated and 1,487 were downregulated (Figure 3A). Furthermore, treatment with MA resulted in alterations in 203 genes compared to the model group, with 3 genes showing upregulation and 200 genes showing downregulation (Figure 3B). Of them, 117 overlapping DEGs between the control vs. model and model vs. MA groups were identified by Venn analysis and visualized using a Heatmap (Figures 3C, D; Supplementary Table S2). To assess the credibility of the CO-DEGs identified in our RNA-Seq analysis, a subset of 25 genes was chosen for validation through qRT-PCR analysis. Among these 25, the expression results of 11 genes (*Pde4b*, *Igf2r*, *Slc6a12*, *Itgb7*, *Tlr9*, *Nox1*, *Mmp12*, *Abca1*, *Cd80*, *Il11*, and *Tnfrsf9*) were consistent with RNA-seq data (Figure 3E). The phosphodiesterases (PDEs) constitute a diverse group of isozymes, with PDE4B being the primary isoform expressed in various inflammatory cells and known to regulate the inflammatory response. Therefore, we focused on PDE4B. Overall, these results suggested that the anti-inflammatory effect on RAW264.7 macrophages of MA may be highly associated with PDE4B function, indicating that MA may exert anti-inflammatory effects by inhibiting the activity of PDE4B.

### 3.3 MA inhibited LPS-induced inflammation via the PDE4B-cAMP-PKA-NF-κB pathway

**FIGURE 4:**
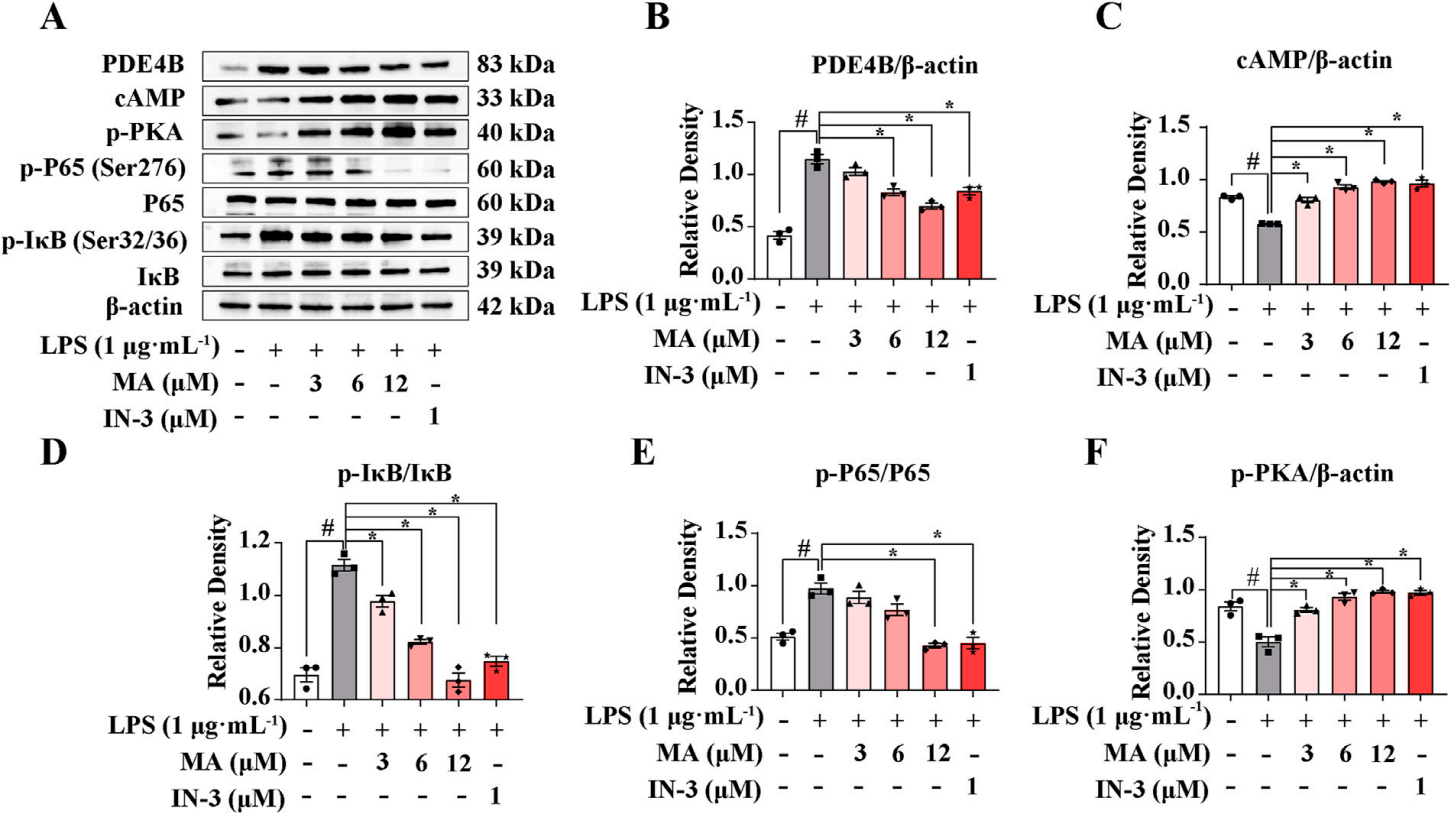
Menisoxoisoaporphine A (MA) inhibited LPS-induced inflammation via PDE4B-cAMP-PKA-NF-κB pathway. RAW264.7 cells were stimulated with 1 μg·mL^-1^ lipopolysaccharide (LPS), and concurrently treated with MA (0, 12 μM) or PDE4B-IN-3 (IN-3, 1 μM) for 12 h. **(A)** Representative Western blot bands and **(B–F)** quantification of PDE4B, cAMP, p-PKA, p-P65(Ser276)/P65 and p-IκB(Ser32/36)/IκB. β-Actin was used as an internal reference. Each value was expressed as the means ± SEM (n = 3). ^#^ *p* < 0.05: vs*.* control group. *p < 0.05: vs*.* only LPS-treated group.

Utilizing transcriptome data, we conducted a detailed analysis of the molecular mechanisms involved in the regulation of PDE4B by MA. It is well known that PDE4B exhibits a high degree of specificity in degrading cAMP, and the increase in cAMP levels resulting from the use of PDE4B inhibitors plays a significant role in inhibiting the release of inflammatory factors from macrophage cells. Increased concentrations of cAMP have been correlated with the stimulation of PKA and the suppression of NF-κB, thus establishing the PDE4B-cAMP-PKA-NF-κB signaling cascade. PDE4B-IN-3, an inhibitor of PDE4B, is used as the positive drug. As shown in Figures 4A, B, PDE4B was upregulated by 2 fold in response to LPS in RAW264.7 cells but significantly decreased following MA treatment (*p* < 0.05). We found that LPS-induced levels of cAMP and p-PKA were significantly reduced compared with the control group, while these proteins were all dose-dependently increased in response to MA treatment (*p* < 0.05) (Figures 4A, C, D). Moreover, the phosphorylation of IκB and P65 was markedly induced by LPS but significantly inhibited by MA treatment (*p* < 0.05) (Figures 4A, E, F). Collectively, these results indicated that MA alleviates the LPS-triggered inflammatory response in RAW264.7 cells through the PDE4B-cAMP-PKA-NF-κB axis. This study not only offers novel insights into the anti-inflammatory mechanisms of MA, but also establishes a theoretical foundation and provides empirical support for the application of MA and analogous compounds in the treatment of inflammatory diseases.

### 3.4 MA inhibited cAMP-PKA-NF-κB-mediated inflammation via PDE4B

**FIGURE 5:**
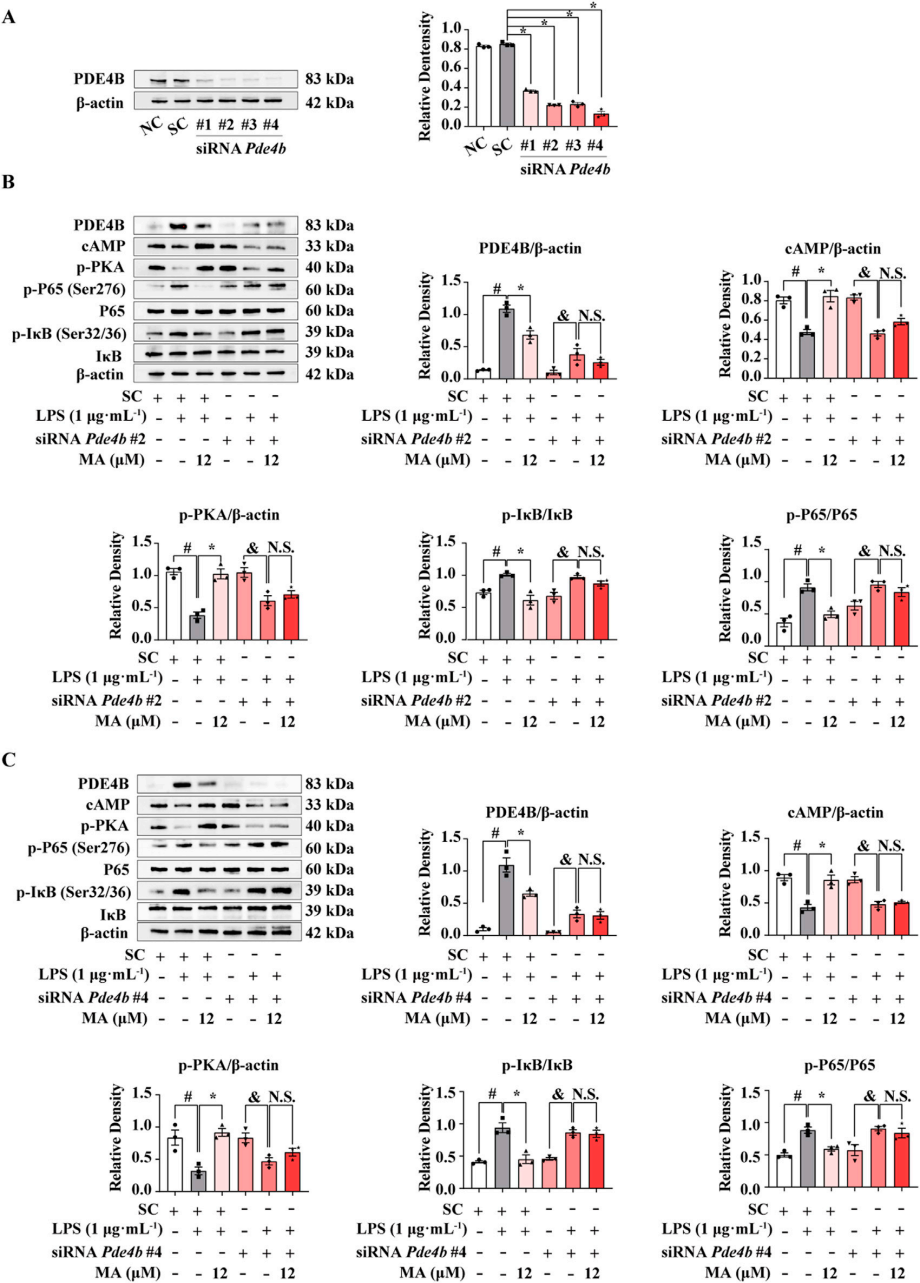
PDE4B knockdown abolished the anti-inflammatory effects of Menisoxoisoaporphine A (MA) at LPS-induced RAW264.7 cells. RAW264.7 cells were transfected with *Pde4b* siRNA or control siRNA for 12 h. **(A)** The PDE4B proteins were probed by western bolt analysis. Transfected cells were then stimulated with 1 μg·mL^−1^ lipopolysaccharide (LPS), and concurrently treated with MA (0, 12 μM) for 12 h. **(B)** Representative Western blot banding pictures and quantification of PDE4B, cAMP, p-PKA, p-IκB(Ser32/36)/IκB and p-P65(Ser276)/P65 of *Pde4b* siRNA#2 treatment. **(C)** Representative Western blot banding pictures and quantification of PDE4B, cAMP, p-PKA, p-IκB(Ser32/36)/IκB and p-P65(Ser276)/P65 of *Pde4b* siRNA#4 treatment. β-Actin was used as an internal reference. NC, cells without any treatment. SC, cells only treated with control siRNA. Each value was expressed as the means ± SEM (n = 3). ^#^ *p* < 0.05: vs*.* control siRNA group. *p < 0.05: vs*.* indicated group.

To confirm the role of PDE4B activation in the effects of MA, we blocked PDE4B activation with siRNA-*Pde4b*. Knocking down PDE4B expression with four different siRNAs led to a robust decrease in the PDE4B protein levels (*p* < 0.05) (Figure 5A). Then, we selected siRNA-*Pde4b #2* and siRNA-*Pde4b #4* with the strongest knockdown efficiency for subsequent experiments. Both siRNA-*Pde4b #2* and siRNA-*Pde4b #4* reversed the protective effects of MA on LPS-induced the inhibition of the cAMP/PKA pathway and activation of the NF-κB signaling pathway in RAW264.7 cells (Figures 5B, C).

**FIGURE 6:**
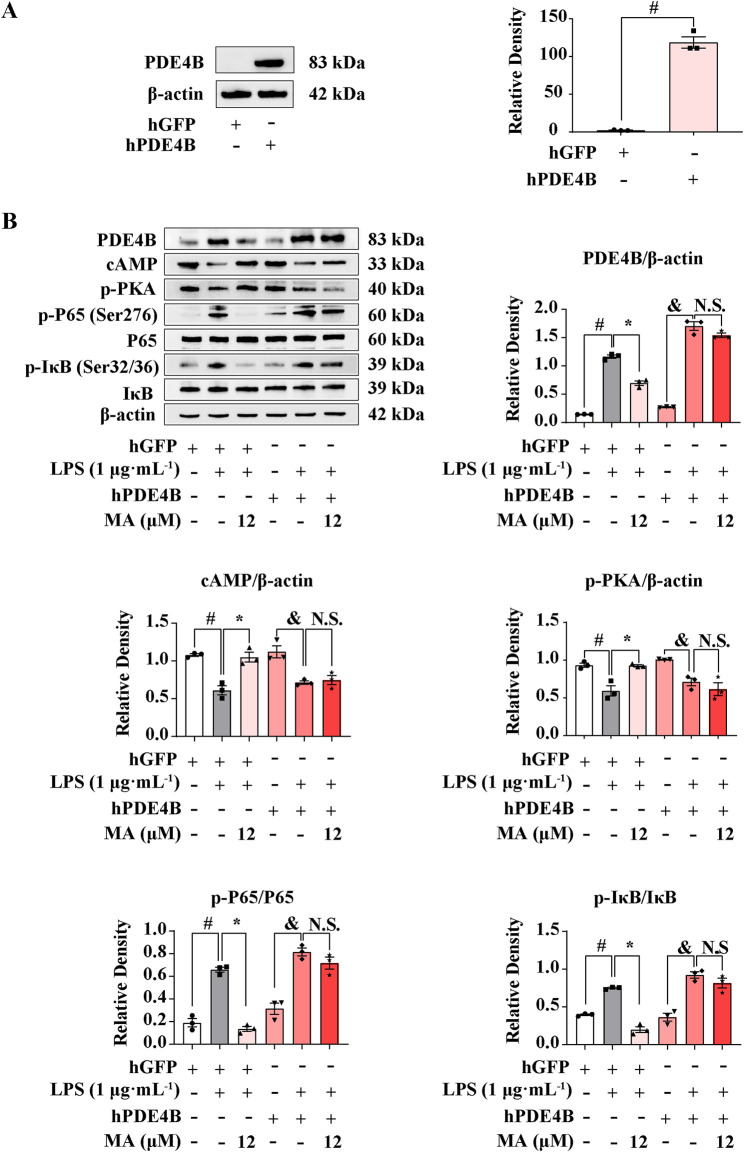
Menisoxoisoaporphine A (MA) inhibited cAMP/PKA-NF-κB via PDE4B. RAW264.7 cells were transfected with pIRES2-EGFP-Pde4b overexpressed plasmid or empty pIRES2-EGFP for 12 h. **(A)** PDE4B protein expression detected by western bolt. Transfected cells were then stimulated with 1 μg·mL^−1^ lipopolysaccharide (LPS), and concurrently treated with MA (0, 12 μM) for 12 h. **(B)** Representative Western blot bands and quantification of PDE4B, cAMP, p-PKA, p-P65(Ser276)/P65 and p-IκB(Ser32/36)/IκB. β-Actin was used as an internal reference. Each value was expressed as the means ± SEM (n = 3). ^#^ *p* < 0.05: vs*.* control hGFP group. *p < 0.05: vs*.* indicated group.

To clarify whether MA exerts an anti-inflammatory effect depending on PDE4B, we performed a PDE4B overexpression experiment on RAW264.7 cells. After transfecting the pIRES2-EGFP-PDE4B plasmid for 12 h, PDE4B expression increased significantly compared to the control group (*p* < 0.05) (Figure 6A). MA treatments significantly inhibited the PDE4B-cAMP-PKA-NF-κB signaling pathway, while PDE4B overexpression abolished the effect of MA on the cAMP-PKA pathway (*p* < 0.05) (Figure 6B). Moreover, the suppressive effect of MA on the NF-κB pathway was no longer observed upon the overexpression of PDE4B, which means that the ameliorating effects of MA on LPS-induced inflammation were abolished in RAW264.7 cells transfected with a PDE4B overexpression plasmid. These findings collectively suggest that MA suppresses inflammation mediated by the cAMP-PKA-NF-κB pathway through its interaction with PDE4B. This interaction may facilitate the development of novel therapeutic agents targeting diseases associated with PDE4B activity.

### 3.5 MA could directly bind to PDE4B at the Tyr405 site

**FIGURE 7:**
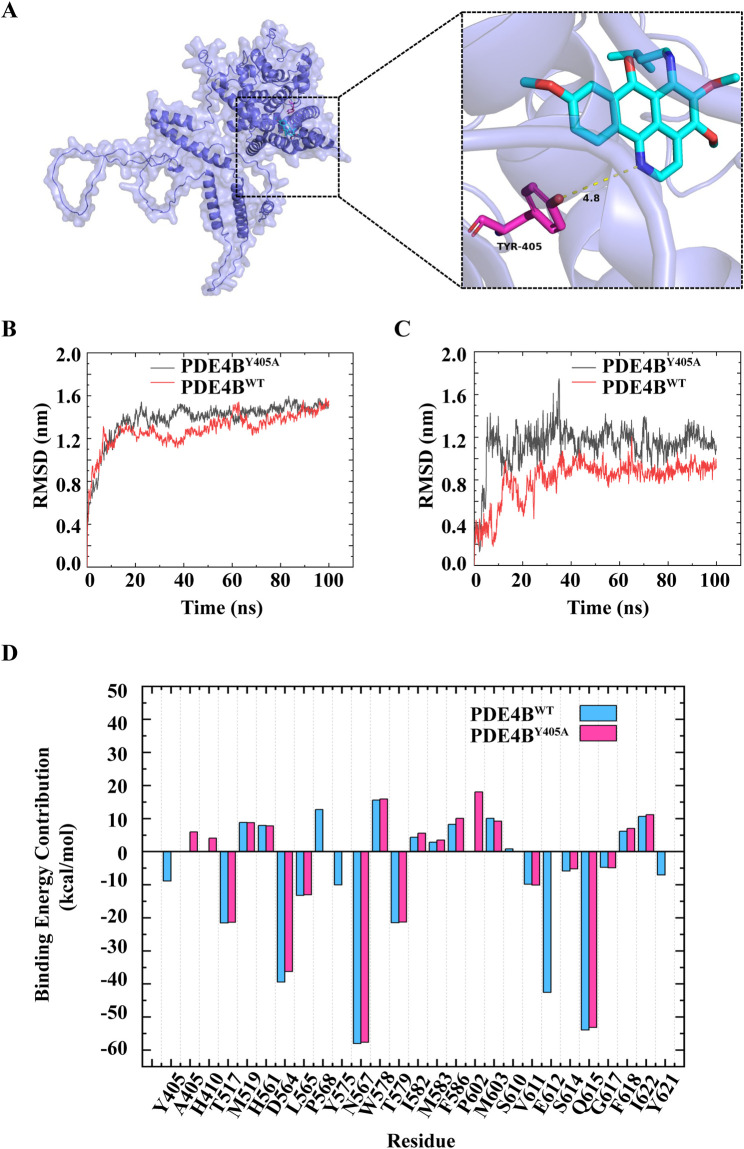
Menisoxoisoaporphine A (MA) could directly bind to PDE4B at Tyr405 site. **(A)** Molecular docking simulation between MA and PDE4B. **(B)** The root mean square deviation (RMSD) of PDE4B in the PDE4B^WT^ system and PDE4B^Y405A^ system. **(C)** The RMSD of MA in the PDE4B^WT^ system and PDE4B^Y405A^ system. **(D)** The residual energy decomposition of the binding free energy of PDE4B^WT^ system and PDE4B^Y405A^ system, the horizontal axis denotes the respective amino acids associated with each position. WT: wild type; TYR: tyrosine; Y405A: The tyrosine residue at position 405 is mutated to glycine.

In order to probe whether MA is directly combined with PDE4B, we performed molecular docking experiments. Based on the PDE4B model, we docked MA using AutoDock. As shown in Figure 7A, stable hydrogen bonds between PDE4B and MA were found. MA could interact with the Tyr405 residue of PDE4B, and the bond length was found to be 4.8 Å. The calculated free energy of the interaction between MA and PDE4B was determined to be −8.2 kcal/mol, suggesting a favorable binding affinity. To further understand the interacting process of MA and PDE4B, we then conducted MD simulation experiments using the native (PDE4B^WT^) and mutant protein (PDE4B^Y405A^) structures. The conformational stability of the PDE4B-MA complexes was assessed by measuring the root mean square deviation (RMSD) values. The RMSD profile showed that PDE4B^WT^ and PDE4B^Y405A^ reached the equilibrium phase at 20 ns for MA (Figure 7B). As shown in Figure 7C, the RMSD of MA in the PDE4B^WT^ system was smaller than that in the PDE4B^Y405A^ system, indicating a decrease in the stability of MA after the Tyr405 site mutation of the PDE4B protein. Due to the system stabilizing after 20 ns, we selected trajectories in the range of 20–100 ns for the MM-PBSA binding free energy calculation. According to the data presented in Table 3, the binding free energy of the MA-PDE4B^WT^ system was lower than that of the MA-PDE4B^Y405A^, indicating that MA-PDE4B^WT^ system is more stable. In order to conduct an in-depth examination of the interplay between MA and PDE4B, we analyzed the binding free energy of MA-PDE4B^WT^ and MA-PDE4B^Y405A^ complexes to assess the individual energy contributions of each protein residue, thereby identifying the critical binding residues. The results showed that in the MA-PDE4B^WT^ system, Tyr405 showed a favorable binding effect (−8.88 kcal/mol), while in the MA-PDE4B^Y405A^ system, Ala405 demonstrated an unfavorable binding effect (5.92 kcal/mol) (Figure 7D). These results indicated that the mutation of Tyr405 has a critical impact on the stability of MA-PDE4B. Overall, the findings suggested that MA could directly bind to the PDE4B Tyr405 site, indicating a potential mechanism through which MA may influence inflammation, immune function, and cardiovascular processes. Selective PDE4B inhibitors could provide benefits over non-selective PDE4 inhibitors by minimizing off-target effects and enhancing safety profiles.

**TABLE 3:** Binding free energy and different energy contributions of MA-PDE4B^WT^ systems and MA-PDE4B^Y405A^ systems (kcal/mol).

Complexe	△Eele	△EvdW	△GP	△Gnp	△Egas	△Esol	△Gbind
MA-PDE4B^WT^	−4.02	−47.68	26.39	−4.65	−51.70	21.74	−29.96
MA-PDE4B^Y405A^	−1.51	−40.93	23.64	−4.28	−42.43	19.35	−23.08

## 4 Discussion

Although inflammation is generally thought of as a defense against various attacks, excessive inflammatory reactions can lead to serious diseases, including but not limited to asthma, atherosclerosis, arthritis, and diabetes (Furman et al., 2019). Multiple studies have shown that inhibiting the inflammatory response is a critical component of therapeutic strategies for various inflammatory diseases (Wang et al., 2020; Bhattarai et al., 2016; Yang et al., 2022). Because of the side effects in the use of anti-inflammatory drugs clinically, there is a pressing demand for novel anti-inflammatory drugs to effectively address inflammatory diseases. Currently, natural products serve as a potential reservoir of anti-inflammatory agents that have yet to be fully explored (Dinarello, 2010; Wu et al., 2023).

Beidougen is recognized for its therapeutic properties in clearing heat, detoxifying the body, dispelling wind, and alleviating pain (Chinese Pharmacopoeia Commission, 2020). It is commonly utilized in traditional medicine for the treating inflammatory conditions such as enteritis, dysentery, rheumatism, and bronchitis (Anon, 1977). Tablets and capsules containing the total alkaloids of Beidougen have been extensively utilized in clinical settings for the treatment of pharyngitis, tonsillitis, and chronic bronchitis (Su et al., 2015). Modern research also has found that the alkaloids of Beidougen have notable anti-inflammatory properties. It has been shown to possess inhibitory effects on croton oil-induced auricular edema in mice and carrageenan-induced plantar edema in rats (Du et al., 1987). The administration of dauricine has been shown to effectively ameliorate severe pneumonia induced by avian influenza virus H5N1 and *Streptococcus* pneumoniae in mice (Li et al., 2018). Our present study demonstrates, for the first time to our knowledge, that MA, a novel compound isolated from Beidougen, plays an anti-inflammatory role by targeting PDE4B in LPS-induced RAW264.7 macrophage cells.

PDEs are a family of enzymes that catalyze cyclic nucleotides, which consists of 11 structurally related and functionally distinct PDE gene families (PDE1-11) (Essayan, 2001). Of the 11 PDE families, PDE4, PDE7, and PDE8 exhibit selectivity in catalyzing the hydrolysis of cAMP to AMP (Azevedo et al., 2014). cAMP is a critical intracellular second messenger that activates PKA, which inhibits the phosphorylation of the p65 subunit of NF-κB to inactivate NF-κB target genes such as *Nos2*, *Tnf-α*, and *Il-6* (Yan et al., 2022). PDE4 is highly expressed in most cells and is the main cAMP-specific PDE (Beghè et al., 2013). The PDE4 family consists of PDE4A, PDE4B, PDE4C, and PDE4D, which exhibit high levels of expression in inflammatory immune cells, particularly macrophages (Crilly et al., 2011; Schafer, 2012). PDE4B has been demonstrated to be upregulated by various inflammatory stimuli, which play a key role in mediating an inflammatory response (Jin et al., 2010; Huang et al., 2019). Based on the transcriptomic data, we indeed found that *Pde4b* gene was highly expressed in LPS-induced RAW264.7 cells. Meanwhile, the levels of cAMP and p-PKA were significantly decreased by LPS alone. Led by this observation, we investigated the signaling pathway downstream of p-PKA and concluded that the NF-κB pathway is in an activated state. An increasing body of research indicates that targeting PDE4 may be a promising approach for treating inflammatory diseases, which has lead to the gradual development of selective PDE4 inhibitors (Li et al., 2018; Li et al., 2022; Schepers et al., 2023). Phase I and II clinical trials treating patients with idiopathic pulmonary fibrosis using a novel PDE4B inhibitor, BI 1015550, have shown promising results (Richeldi et al., 2022). Our studies showed that MA exerted a significant inhibitory effect on PDE4B protein expression. To further investigate the underlying mechanisms of MA against the LPS-induced inflammatory model, we performed a transwell assay using RAW264.7 cells with either PDE4B overexpression or knockdown. We found that overexpression of PDE4B significantly reversed the protective effect of MA, as evidenced by decreased levels of cAMP and p-PKA, along with increased levels of p-IκB and p-P65. Likewise, knockdown of PDE4B or MA increased cAMP and p-PKA and downregulated the expression of p-IκB and p-P65, indicating that MA inhibited cAMP-PKA-NF-κB-mediated inflammation responses depending on PDE4B. Further, molecular docking studies and molecular dynamic simulation assays jointly indicated that MA could directly bind to PDE4B at Tyr405 site, showing a great potential to be developed into an effective selective PDE4B inhibitor.

**FIGURE 8:**
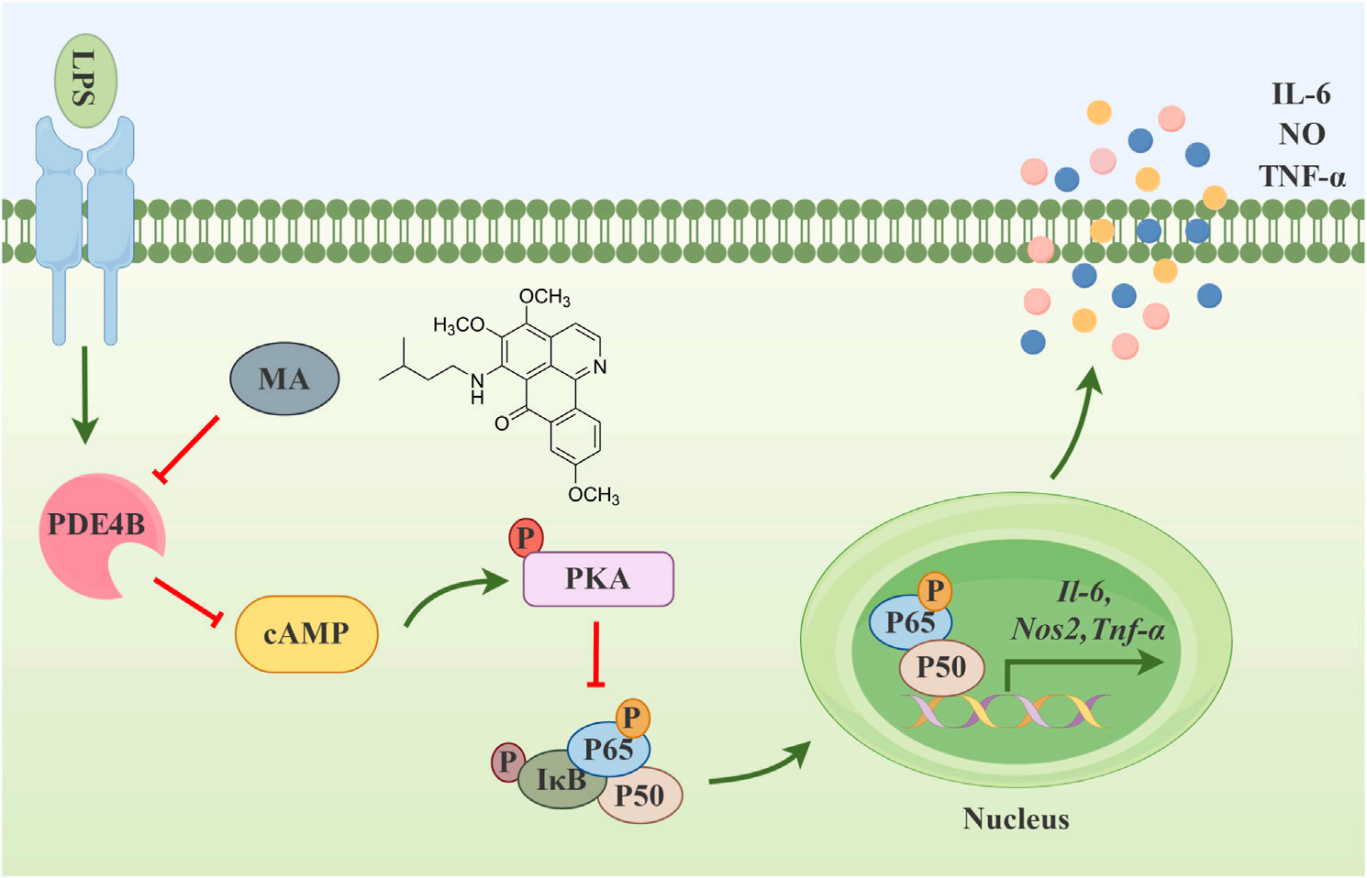
Menisoxoisoaporphine A (MA) inhibited cAMP-PKA-NF-κB mediated inflammation via targeting PDE4B.

In conclusion, the significant findings of this study are as follows: First, MA had a significantly inhibitory effect on the LPS-induced inflammatory response in RAW264.7 cells. The administration of 3 μM MA exhibits superior efficacy in inhibiting inflammatory factors relative to 12 μM dexamethasone in RAW264.7 cells stimulated with 1 μg·mL^-1^ LPS, while its anti-inflammatory activity is comparable to that of 25 μM isoquercetin (Bai et al., 2022; Luan et al., 2022). Second, MA could specifically act at the Tyr405 site of PDE4B, inhibiting the production of PDE4B and blocking downstream NF-κB signaling pathways via the cAMP-PKA axis. The upstream regulatory pathway of the NF-κB signaling cascade encompasses a multitude of intricate molecular mechanisms and signaling events, which include cytokines (e.g., TNF-α, IL-1), pathogen-associated molecular patterns (e.g., LPS), damage-associated molecular patterns (e.g., ATP) and their corresponding receptors. Nonetheless, In our study, the regulation of NF-κB was specifically mediated via the phosphorylation of PKA (Imran et al., 2023; Sajid et al., 2023; Sajid et al., 2021). Third, MA inhibited the cAMP-PKA-NF-κB-mediated inflammatory response dependent on PDE4B (Figure 8, By Figdraw). Thus, we have discovered a new anti-inflammatory compound, MA, which may serve as a promising therapeutic option for the management of inflammatory conditions through its targeting of PDE4B. By targeting PDE4B with MA, researchers may be able to develop therapies that specifically downregulate inflammatory pathways, thereby mitigating the symptoms and progression of various inflammatory diseases. For example, in chronic obstructive pulmonary disease, the inhibition of PDE4B may result in a reduction of neutrophilic inflammation and mucus hypersecretion, both of which are characteristic features of the disease (Lim et al., 2021). Similarly, targeting PDE4B in acute myocardial infarction may reduce infarct size and protect microvessels by regulating neutrophil inflammatory response (Wan et al., 2022). Furthermore, MA’s specific inhibition of PDE4B may reduce off-target effects, a common issue in drug development. This specificity could lead to better safety and patient outcomes. However, it is still unclear whether MA only has an inhibitory effect on PDE4B. Based on transcriptome data, we did not found that MA had inhibitory effects on other genes in the PDE family. Consequently, we intend to further investigate whether MA has strong selectivity in inhibiting PDE4B. In addition, due to the fact that MA is a new compound and the amount is relatively small, we are currently unable to investigate the effect of MA on inflammatory animal models. Currently, we are addressing the challenge of synthesizing MA. Therefore, inspired by the findings above, we are interested in further exploring the administration, dosage, efficacy, long-term safety, and tolerability of MA in various animal models of inflammation, assessing its pharmacokinetics and pharmacodynamics. Building on the findings from animal experiments, we intend to design preliminary clinical trials to assess the safety and efficacy of MA in human subjects, ultimately paving the way for its potential use in the clinic.

## Data Availability

The datasets presented in this study can be found in online repositories. The names of the repository/repositories and accession number(s) can be found in the article/Supplementary Material.
