# Supplementary material for: Menisoxoisoaporphine A, a novel oxoisoaporphine alkaloid from Menispermi Rhizoma, inhibits inflammation by targeting PDE4B
Source: Front Pharmacol. 2024 Dec 3;15:1505116. doi: 10.3389/fphar.2024.1505116 (PMC11649434; doi:10.3389/fphar.2024.1505116)
Supplement: Supplementary file 1 [file Table1.DOCX]

Supplementary Material

Menisoxoisoaporphine A, a novel oxoisoaporphine alkaloid from Menispermi Rhizoma, inhibits inflammation by targeting PDE4B

Xin Qiao^†,1,2,3^, Xiaojuan Cao^†,1,2,3^, Shuang Xu^2,3,4^, Cunlin Wang^1,2,3^, Rui Guo^1,2,3^, Xiaojuan Yao^1,2,3^, Qiong Zhang ^1,2,3,*^

*****Correspondence: [Zhangqiong@sxmu.edu.cn](mailto:Zhangqiong@sxmu.edu.cn)

†These authors contributed equally to this work and share first authorship

**Table S1.** Primary antibodies used in this study

| Antibody | Dilution | Supplier | Reference |
| --- | --- | --- | --- |
| PDE4B | 1:1000 | Abcam | #ab170939 |
| cAMP | 1:20000 | Abcam | #ab76238 |
| p-PKA | 1:1000 | Abcam | #ab75991 |
| β-actin | 1:10000 | Bioworld | #BS6007M |
| P65 | 1:1000 | Affinity | #[AF5006](https://www.scbt.com/zh/p/zo-1-antibody-r40-76?requestFrom=search) |
| p-P65(Ser276) | 1:1000 | Beyotime | #AF5875 |
| IκB | 1:1000 | Affinity | #AF5002 |
| p-IκB(Ser32/36) | 1:1000 | Beyotime | #AF5851 |

**Table S2.** The details of 117 differential expression genes (DEGs).

| Gene ID | Gene Name | Log2（B/A） | Log2（C/B） | Gene ID | Gene Name | Log2（B/A） | Log2（C/B） |
| --- | --- | --- | --- | --- | --- | --- | --- |
| 104886 | *Rab15* | 6.66 | -1.84 | 19261 | *Sirpa* | -0.74 | -0.59 |
| 108115 | *Slco4a1* | 1.40 | -0.79 | 19411 | *Rarg* | -0.69 | -0.92 |
| 110920 | *Hspa13* | 0.68 | -0.82 | 20210 | *Saa3* | 4.21 | -0.83 |
| 11303 | *Abca1* | 4.33 | -5.18 | 20249 | *Scd1* | -0.82 | -0.67 |
| 11487 | *Adam10* | -0.88 | -0.64 | 20250 | *Scd2* | -1.18 | -0.95 |
| 11540 | *Adora2a* | 6.99 | -1.31 | 20299 | *Ccl22* | 10.74 | -0.78 |
| 11541 | *Adora2b* | 2.86 | -0.59 | 20352 | *Sema4b* | 1.83 | -1.14 |
| 11977 | *Atp7a* | 0.65 | -0.62 | 20353 | *Sema4c* | 2.41 | -0.78 |
| 12125 | *Bcl2l11* | 2.45 | -0.59 | 20660 | *Sorl1* | -1.83 | -0.71 |
| 12226 | *Btg1* | 1.50 | -0.74 | 20706 | *Serpinb9b* | 4.05 | -0.73 |
| 12273 | *C5ar1* | 1.27 | -0.60 | 20750 | *Spp1* | 0.65 | -0.77 |
| 12475 | *Cd14* | 3.02 | -0.78 | 208647 | *Creb3l2* | 1.51 | -0.77 |
| 12519 | *Cd80* | 2.68 | -1.07 | 216856 | *Nlgn2* | 2.21 | -0.67 |
| 12522 | *Cd83* | 4.51 | -1.12 | 21807 | *Tsc22d1* | 1.58 | -0.87 |
| 12628 | *Cfh* | -1.95 | -1.14 | 21923 | *Tnc* | 6.28 | -2.98 |
| 12822 | *Col18a1* | -0.97 | -0.66 | 21938 | *Tnfrsf1b* | 3.54 | -0.77 |
| 12874 | *Cpd* | 1.89 | -0.77 | 21942 | *Tnfrsf9* | 7.13 | -1.89 |
| 12916 | *Crem* | 1.21 | -0.63 | 225372 | *Apbb3* | 2.21 | -0.68 |
| 12984 | *Csf2rb2* | 1.23 | -0.80 | 225638 | *Alpk2* | 6.79 | -1.37 |
| 13051 | *Cx3cr1* | -0.76 | -0.59 | 226519 | *Lamc1* | 0.70 | -1.00 |
| 13058 | *Cybb* | 0.79 | -0.66 | 230752 | *Eva1b* | 5.29 | -0.77 |
| 13612 | *Edil3* | 2.29 | -1.29 | 237038 | *Nox1* | 5.13 | -1.28 |
| 13733 | *Adgre1* | 1.67 | -0.65 | 257632 | *Nod2* | 2.56 | -0.61 |
| 14102 | *Fas* | 3.86 | -0.63 | 26364 | *Adgre5* | -1.77 | -0.98 |
| 14204 | *Il4i1* | 1.87 | -1.16 | 26570 | *Slc7a11* | 4.38 | -0.72 |
| 14411 | *Slc6a12* | 1.92 | -1.69 | 27029 | *Sgsh* | -1.25 | -0.59 |
| 14425 | *Galnt3* | 1.27 | -0.62 | 27362 | *Dnajb9* | 1.79 | -0.60 |
| 14609 | *Gja1* | -0.70 | -0.66 | 319772 | *C130050O18Rik* | -1.73 | -0.83 |
| 15370 | *Nr4a1* | 1.21 | -1.45 | 320581 | *Idi2* | 5.33 | -5.27 |
| 15567 | *Slc6a4* | 5.20 | -0.81 | 321019 | *Gpr183* | -0.91 | -0.63 |
| 15937 | *Ier3* | 3.54 | -0.64 | 327766 | *Tmem26* | 2.50 | -1.21 |
| 16004 | *Igf2r* | 2.10 | -1.63 | 328232 | *Gfod1* | 1.36 | -0.83 |
| 16156 | *Il11* | 7.78 | -2.45 | 381677 | *Vgf* | 3.09 | -0.73 |
| 16176 | *Il1b* | 11.66 | -1.32 | 434215 | *Lrrc32* | -3.76 | -1.35 |
| 16193 | *Il6* | 11.41 | -1.23 | 50764 | *Fbxo15* | 2.48 | -0.98 |
| 16401 | *Itga4* | -0.62 | -0.73 | 51902 | *Rnf24* | 1.73 | -0.73 |
| 16408 | *Itgal* | 1.53 | -0.71 | 53322 | *Nucb2* | 0.67 | -0.66 |
| 16411 | *Itgax* | 2.50 | -1.38 | 53421 | *Sec61a1* | 0.64 | -0.61 |
| 16421 | *Itgb7* | 1.60 | -1.00 | 53618 | *Fut8* | 1.12 | -0.63 |
| 16449 | *Jag1* | 1.69 | -1.01 | 54216 | *Pcdh7* | 2.02 | -0.66 |
| 16728 | *L1cam* | -1.52 | -1.05 | 54338 | *Slc23a2* | 0.66 | -0.79 |
| 16835 | *Ldlr* | 0.77 | -0.72 | 54448 | *Il1f6* | 6.75 | -2.25 |
| 16878 | *Lif* | 7.27 | -1.44 | 56338 | *Txnip* | -0.68 | -0.69 |
| 16918 | *Mycl* | 6.61 | -1.39 | 56434 | *Tspan3* | 1.08 | -0.59 |
| 170776 | *Cd209c* | -0.60 | 0.61 | 67972 | *Atp2b1* | 0.97 | -0.64 |
| 17381 | *Mmp12* | 3.43 | -1.48 | 70350 | *Basp1* | 8.14 | -1.25 |
| 17387 | *Mmp14* | 6.43 | -1.65 | 71914 | *Antxr2* | 1.78 | -0.67 |
| 18128 | *Notch1* | 1.29 | -1.39 | 72027 | *Slc39a4* | 1.58 | -0.62 |
| 18129 | *Notch2* | 0.60 | -1.06 | 73827 | *Tmem198b* | 1.09 | -0.71 |
| 18186 | *Nrp1* | -0.82 | -0.93 | 74126 | *Syvn1* | 0.86 | -0.72 |
| 18187 | *Nrp2* | 2.05 | -1.02 | 74309 | *Osbp2* | 9.62 | -2.31 |
| 18227 | *Nr4a2* | 1.20 | -1.78 | 75202 | *Spaca6* | 3.18 | -1.04 |
| 18578 | *Pde4b* | 1.94 | -0.64 | 75767 | *Rab11fip1* | 2.38 | -0.67 |
| 18844 | *Plxna1* | 0.84 | -0.63 | 78892 | *Crispld2* | 4.68 | -5.58 |
| 18858 | *Pmp22* | 1.33 | -0.69 | 79202 | *Tnfrsf22* | 1.01 | -0.67 |
| 19124 | *Procr* | 5.17 | -0.76 | 81897 | *Tlr9* | 1.20 | -1.11 |
| 19204 | *Ptafr* | 1.35 | -0.62 | 83382 | *Siglece* | 1.45 | -0.85 |
| 19225 | *Ptgs2* | 6.37 | -1.42 | 83397 | *Akap12* | 4.41 | -2.20 |
| 94219 | *Cnnm2* | 0.96 | -1.01 |  |  |  |  |

Note: A, B and C represent the average FPKM values of control, model and MA group respectively.
